# Micronutrients and Leptospirosis: A Review of the Current Evidence

**DOI:** 10.1371/journal.pntd.0004652

**Published:** 2016-07-07

**Authors:** Heather S. Herman, Saurabh Mehta, Washington B. Cárdenas, Anna M. Stewart-Ibarra, Julia L. Finkelstein

**Affiliations:** 1 Division of Nutritional Sciences, Cornell University, Ithaca, New York, United States of America; 2 St. John's Research Institute, Bangalore, India; 3 Laboratorio de Biomedicina, Escuela Superior Politécnica del Litoral, ESPOL, Guayaquil, Ecuador; 4 Department of Medicine and the Center for Global Health and Translational Science, State University of New York (SUNY) Upstate Medical University, Syracuse, New York, United States of America; Mahidol University, THAILAND

## Abstract

**Background:**

Leptospirosis is one of the most widespread zoonoses and represents a major threat to human health. Due to the high burden of disease, limitations in diagnostics, and limited coverage and availability of effective human and veterinary vaccines, leptospirosis remains an important neglected zoonotic disease. Improved surveillance and identification of modifiable risk factors for leptospirosis are urgently needed to inform preventive interventions and reduce the risk and severity of *Leptospira* infection.

**Methodology/Principal Findings:**

This review was conducted to examine the evidence that links micronutrient status and *Leptospira* infection. A total of 56 studies were included in this review: 28 in vitro, 17 animal, and 11 observational human studies. Findings indicated that *Leptospira* infection is associated with higher iron and calcium concentrations and hypomagnesemia.

**Conclusions/Significance:**

Few prospective studies and no randomized trials have been conducted to date to examine the potential role of micronutrients in *Leptospira* infection. The limited literature in this area constrains our ability to make specific recommendations; however, the roles of iron, calcium, and magnesium in leptospirosis represent important areas for future research. The role of micronutrients in leptospirosis risk and severity needs to be elucidated in larger prospective human studies to inform public health interventions.

## Introduction

Leptospirosis is a widespread zoonotic disease endemic in tropical and subtropical regions, caused by infection with pathogenic bacteria of the genus *Leptospira* [1,2]. Humans become infected with leptospirosis through contact with urine of infected animals or with contaminated water in the environment [3]. Socioeconomic and environmental factors, such as inadequate sanitation and hygiene, lack of potable water, flooding, and close contact with livestock (e.g., fishing, farming) increase the risk of transmission.

Leptospirosis poses a major threat to human health and development, with an estimated 873,000 infections and 48,000 deaths annually [4]. The actual burden of leptospirosis may be even higher than current estimates [5], due to limited active surveillance and common misdiagnosis with febrile illnesses with similar clinical presentation (i.e., fever, myalgia, and headache), such as malaria, dengue fever, typhoid, and chikungunya [3,6]. Gold standard diagnostic methods (i.e., microscopic agglutination test [MAT] and polymerase chain reaction [PCR]) are not widely available in resource-limited settings with the highest burden of disease [7,8], and commercially available rapid tests for leptospirosis exhibit lower sensitivity and specificity. Although veterinary vaccines are available, they require serovar specificity and only cover a few strains of *Leptospira*. Human vaccinations are available in only a few countries (e.g., China, Cuba), and are not widely available in resource-limited settings with the highest burden [3]. Due to the high burden of disease, limitations in diagnostics, and limited coverage and availability of effective vaccines, leptospirosis remains an important neglected zoonotic disease. The World Health Organization (WHO) recently established the Leptospirosis Burden Epidemiology Reference Group (LERG) [5] to develop accurate estimates of leptospirosis disease burden and inform the identification of modifiable risk factors and preventive interventions.

The pathogenesis of leptospirosis and host immune response is not fully understood. Rats were identified as the first primary hosts for *Leptospira*, but in recent decades, leptospirosis has been noted in other rodents, dogs, cattle, swine, horses, and sheep. The high prevalence of infection in domesticated animals poses an enormous threat to humans living in close contact and to international travelers who engage in tourism with wildlife [3]. Contact of bodily mucosal membranes or skin abrasions with contaminated water transmits the leptospires, which disseminate through circulation and adhere to proteins of the host extracellular matrix, including collagen, fibronectin, and laminin. During this initial stage, *Leptospira* can be found in circulation for up to two weeks. As the host adaptive immune response begins, leptospires colonize in the proximal renal tubular epithelium of the kidney and can be found in the urine (up to 10^7^ leptospires/mL). The most severe manifestation of *Leptospira* infection, known as Weil’s syndrome, is associated with renal and liver failure; acute renal failure and severe pulmonary hemorrhage syndrome (SPHS) are the leading causes of mortality [9,10]. In humans, early host production of inflammatory mediators and anti-inflammatory cytokines has been shown to prevent progression to severe disease [11]. However, a delayed immune response in susceptible hosts enables leptospire dissemination to multiple organs, contributing to tissue damage and organ failure.

The vicious cycle of malnutrition and infection was first noted over 50 years ago [12], implicating micronutrient deficiencies as both a risk factor and a consequence of infection [13–16]. Micronutrient deficiencies influence innate and adaptive host immune response to infections, including macrophage and lymphocyte function, metabolic functions [17,18], and risk and severity of infectious diseases [19], while infectious diseases may influence micronutrient absorption, metabolism, and biomarkers of host nutritional status [13].

Emerging evidence supports a role of micronutrients in neglected tropical diseases [20]. In a 2012 report on neglected zoonoses, WHO cited nutritional status as an underappreciated, underlying cause of death in zoonotic infections [21]. However, the role of nutrition in *Leptospira* infection has not been established. Improved surveillance, differential diagnostic methods, and identification of modifiable risk factors for leptospirosis are urgently needed to inform preventive interventions and reduce the risk and severity of leptospirosis.

The objective of this review was to examine the evidence that links micronutrients and leptospirosis. We examined data from experimental laboratory studies and observational human studies on the role of micronutrients in risk, transmission, and severity of *Leptospira* infection. We then discuss research gaps and implications of findings for the development of preventive interventions for leptospirosis, with emphasis on resource-limited settings.

## Methods

### Search Strategy and Selection Process

**Fig 1:**
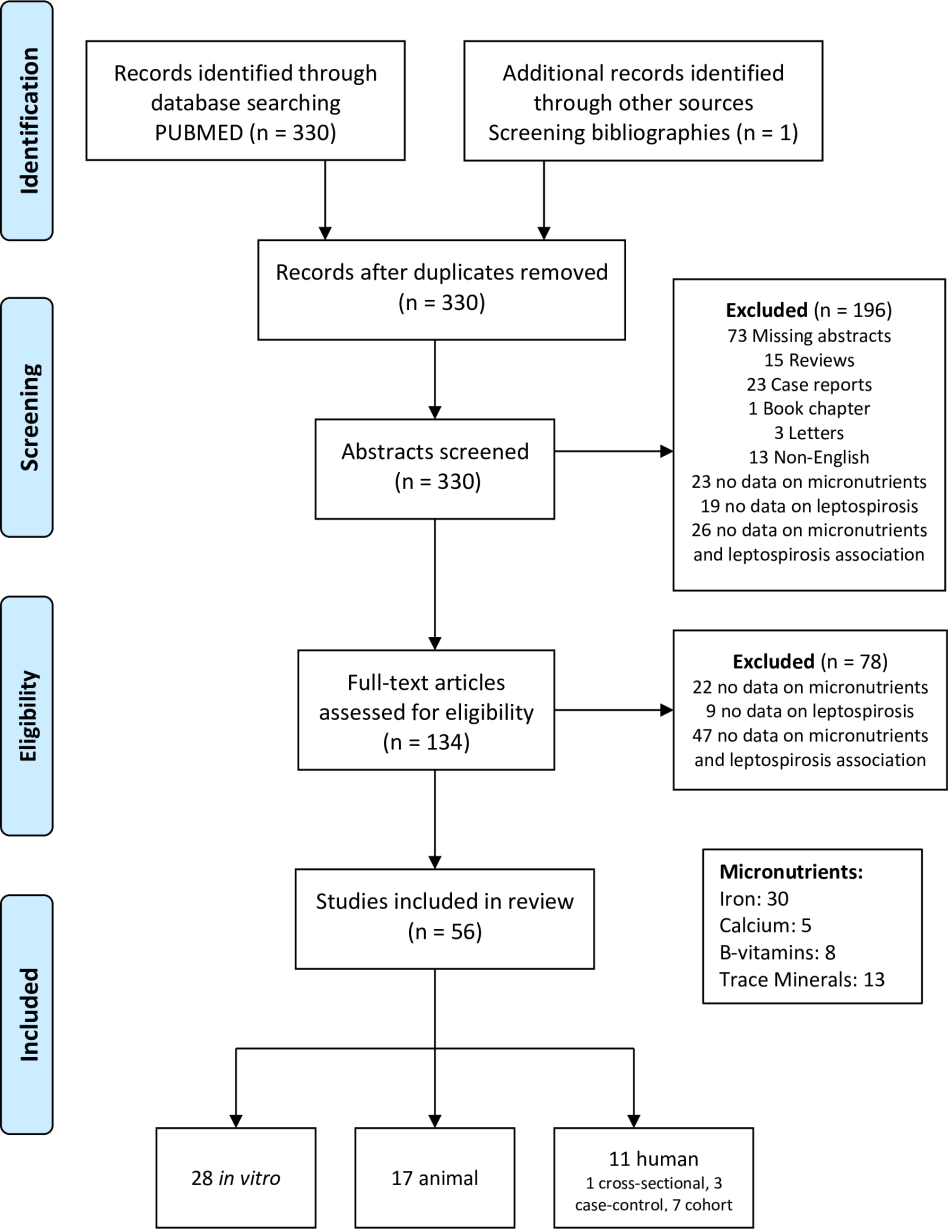
Search strategy: A diagrammatic representation of the retrieval strategy used for identifying and selecting studies for inclusion in the final analysis.

A structured literature search was conducted to examine the associations of micronutrients and leptospirosis using the MEDLINE electronic database. Relevant Medical Subject Heading (MeSH) terms were used to identify published studies. The MeSH search terms included are presented in Box 1. The search strategy is summarized in Fig 1.

**Box 1. Medical Subject Heading (MeSH) Terms**

((((((micronutrients OR micronutrient OR trace element OR trace elements OR vitamins OR vitamin OR carotenoids OR carotenoid OR carotenes OR carotene))) OR (("24,25-dihydroxyvitamin D 3" OR "25-hydroxyvitamin D 2" OR "4- aminobenzoic acid" OR acetylcarnitine OR alpha-tocopherol OR aminobenzoic acids OR ascorbic acid OR beta carotene OR beta-tocopherol OR biotin OR boron OR cadmium OR calcifediol OR calcitriol OR calcium OR Ca2 OR carnitine OR cholecalciferol OR chromium OR cobalt OR cobamides OR cod liver oil OR copper OR dehydroascorbic acid OR dihydrotachysterol OR dihydroxycholecalciferols OR ergocalciferols OR flavin mononucleotide OR folic acid OR formyltetrahydrofolates OR fursultiamin OR gamma-tocopherol OR hydroxocobalamin OR hydroxycholecalciferols OR inositol OR iodine OR iron OR leucovorin OR manganese OR magnesium OR molybdenum OR niacin OR niacinamide OR nickel OR nicotinic acids OR palmitoylcarnitine OR pantothenic acid OR pteroylpolyglutamic acids OR pyridoxal OR pyridoxal phosphate OR pyridoxamine OR pyridoxine OR riboflavin OR selenium OR silicon OR tetrahydrofolates OR thiamine OR thiamine monophosphate OR thiamine pyrophosphate OR thiamine triphosphate OR thioctic acid OR tocopherols OR tocotrienols OR ubiquinone OR vanadium OR zinc))) OR ((Anemia, Iron-Deficiency[Mesh] OR Iron, Dietary[Mesh] OR iron*[tw] OR ferric compounds[Mesh] OR ferrous compounds[Mesh] OR ferrous[tw] OR ferric[tw] OR fe[tw] OR hemoglobin[MeSH] OR hematocrit[MeSH] OR haemoglobin*[tw] OR hemoglobin*[tw] OR anemia[tw] OR anaemia[tw] OR anemic[tw] OR anaemic[tw] OR ferritin)))) AND (("leptospirosis"[MeSH Terms] OR "leptospirosis"[All Fields]) OR ("leptospira"[MeSH Terms] OR "leptospira"[All Fields]) OR leptospires[All Fields] OR "field fever"[All Fields] OR "pretibial fever"[All Fields] OR "Weil's disease"[All Fields] OR "Weil's syndrome"[All Fields] OR "L. icterohemorrhagiae"[All Fields] OR "l. hebdomadis"[All Fields] OR "l. pyrogenes"[All Fields] OR "L. interrogans"[All Fields] OR "l. biflexa"[All Fields])

Initial inclusion criteria for this review were the availability of an abstract and inclusion of data on both micronutrients and leptospirosis. The following micronutrients were considered for inclusion in this review: vitamin A, carotenoids; vitamins C, D, E, and K; B-vitamins thiamin, riboflavin, niacin, pantothenic acid, pyridoxine, inositol, biotin, folate, and cobalamin; and minerals boron, cadmium, calcium, chromium, cobalt, copper, iodine, iron, magnesium, manganese, molybdenum, nickel, selenium, silicon, vanadium, and zinc. Abstracts of all studies were searched, full-text articles of studies were extracted and reviewed, and the following inclusion criteria were applied: (i) micronutrient status, intake, or intervention data, (ii) leptospirosis data, and (iii) data on the associations between micronutrients and leptospirosis. Due to the limited availability of human studies, experimental laboratory animal and in vitro studies were also included. All experimental laboratory studies, observational cross-sectional, case-control, and cohort studies, randomized trials and interventions, and quasi-randomized and uncontrolled trials meeting methodological criteria were included. Sources were retrieved, collected, indexed, and assessed for micronutrient and leptospirosis data. Additional sources were identified from bibliographies of published studies, manual searches of related articles in references, and scientific meeting abstracts. An additional search was used to find review articles, which were examined to cross-reference other relevant studies. A standardized data table was used to extract and summarize key information from experimental and observational studies that met the above-mentioned selection criteria. As part of this protocol, publication date, authors, study design, setting, population or sample, definitions of exposures and outcomes, main findings, and study limitations were recorded.

## Results

The structured literature search resulted in 331 articles, which were reviewed for potential inclusion (Fig 1). After screening bibliographies from extracted articles, one additional study was included. After 197 studies were excluded (*n* = 73 missing abstracts, *n* = 15 literature reviews, *n* = 23 case reports, *n* = 1 book chapter, *n* = 3 editorials or letters to the editor, *n* = 23 no data on micronutrients, *n* = 19 no data on leptospirosis, *n* = 26 no data on the association between micronutrients and leptospirosis, *n* = 13 non-English languages, *n* = 1 duplicate article), 134 full-text articles were extracted for further review. After excluding 78 studies that did not meet the aforementioned inclusion criteria (*n* = 22 no data on micronutrients, *n* = 9 no data on leptospirosis, *n* = 47 no data on the association between micronutrients and leptospirosis), a total of 56 studies were included in this review. These included 45 experimental laboratory studies (28 in vitro and 17 in vivo animal studies) and 11 observational human studies (one cross-sectional, three case-control, and seven cohort studies) (Fig 1). Findings from these studies are summarized in detail in the subsequent supplemental tables (S1–S4 Tables).

### Iron

Iron is required by both the host and pathogen for survival and is a key micronutrient in the context of nutrition, immune function, and infectious diseases. Iron deficiency has been associated with impaired cytokine activity and immune cell proliferation [22] and decreased activity of myeloperoxidase, an iron-dependent enzyme in neutrophils responsible for killing bacteria [14]. The associations between iron and infection are, however, complex and bidirectional. Since pathogens also require iron for survival, host limitation of circulating iron is a defense mechanism as part of the acute phase response; inflammation and cytokine production trigger hepatic synthesis of hepcidin, iron regulation, and sequestering of iron in its storage form, ferritin [23]. *Leptospira* infection may influence iron absorption and metabolism and circulating micronutrient concentrations. Increased serum ferritin concentrations may be attributable to the acute phase response to infection, rather than being a biomarker of actual micronutrient status. Iron status is also influenced by a number of nutritional factors, such as inadequate iron intake and bioavailability, malabsorption, and impaired metabolism. Non-nutritional factors, such as inflammation and infection, also contribute to the etiology of iron deficiency and impact human health [24]. In resource-limited settings, concurrent multiple micronutrient deficiencies and chronic low grade inflammation represent significant challenges and may influence poor health status and higher susceptibility to infectious diseases.

#### In vitro studies

Several laboratory studies have examined the role of iron in leptospiral growth and survival. Iron limitation via addition of iron chelator 2,2-dipyridyl inhibited leptospiral growth in several studies [25–27], while addition of iron in a hemoglobin solution [28,29] and other iron sources (10 μM hemin, 10 μM deferoxamine, and 100 μM of ferric dicitrate) stimulated growth in culture [27].

Other in vitro studies examined the effect of iron limitation on gene transcription. One genome study in *Leptospira biflexa* found putative hemolysins encoded for iron acquisition, and addition of 50 μM 2,2-dipyridyl led to a 10-fold decrease in transcription of ferric uptake regulators [30]. In another in vitro study assessing iron-responsive gene expression in *Leptospira interrogans* serovar *Manilae*, iron limitation conditions (via 40 μM of iron chelator, 2,2’-dipyridyl) down-regulated 49 genes; 16.7% of these genes affected cell division and cell cycle control of leptospires (R^2^ = 0.73, *p* = 0.012) [31]. In a similar study, iron limitation (via 0.4 mM of iron chelator, 2,2-dipyridyl) of *L*. *interrogans* serovar *Copenhageni* up-regulated six proteins involved in bacterial virulence, with a 1.3-fold increase in Loa22 expression, a virulence factor expressed during infection (*p* < 0.05) [32]. Together, these studies suggest that iron limitation affects transcription and expression of genes hypothesized to play a role in infection and pathogenesis.

In a study evaluating the role of iron in *L*. *interrogans* growth rate with a mutant heme oxygenase (HemO) M484, neither wild-type nor M484 *L*. *interrogans* grew in medium lacking iron [33]. Although wild-type *L*. *interrogans* demonstrated rapid growth in a medium supplemented with a solution containing rabbit hemoglobin (*p* < 0.01), the M484 mutant was unable to grow in this medium. Findings indicated that heme oxygenase was required for heme acquisition and growth in an iron source other than FeSO_4_ in standard Ellinghausen-McCullough-Johnson-Harris (EMJH) medium. Together, these findings suggest that heme oxygenase may influence bacterial iron acquisition within the host during *Leptospira* infection.

An in vitro study was conducted to examine the effects of transposon insertion inactivation of *La4131*, a putative outer membrane M48 metalloprotease of *L*. *interrogans* that responds to stress and excessive iron influx [34]. *La4131* inactivation resulted in an 8-fold reduction in transcript levels after the point of insertion, compared to parent type (*p* < 0.01). *La4131* inactivation also resulted in a 2-fold reduction in expression of 11 genes in standard EMJH medium and 13 genes in EMJH supplemented with iron (360 μm FeSO_4_); five of these genes encode proteins for bacterial stress response. Iron-supplemented cultures released outer membrane vesicles and developed an orange precipitate, indicative of potential toxicity. Findings suggest that *L*. *interrogans* requires functional *La4131* metalloprotease for regulation of iron overload on the outer membrane surface, and removal of excess environmental iron is required for bacteria survival.

In another in vitro study, investigators examined the effects of iron and hemoglobin on leptospiral chemotaxis and virulence (i.e., if leptospires flagellate to hosts randomly or toward specific skin abrasions) [35]. Virulent strains of *L*. *interrogans* exhibited significant chemotaxis toward hemoglobin (*p* < 0.01), while avirulent strains of *L*. *interrogans* and saprophytic strains of *L*. *biflexa* did not (*p* > 0.05). Findings indicate an association between chemotaxis to hemoglobin and pathogen virulence, suggesting a potential adaptation for iron acquisition.

Findings from in vitro studies to date demonstrate that iron is required for leptospire growth and survival, although implications for infection and severity are unknown.

#### Animal studies

An animal study in Brazil examined the effects of *L*. *interrogans* serovar *Pomona* infection on iron status in hamsters [36]. Leptospirosis-infected hamsters had significantly higher serum ferritin (*p* < 0.01), serum iron (*p* < 0.01), and hepcidin (*p* < 0.01) concentrations and significantly lower serum transferrin (*p* < 0.05), erythrocyte (*p* < 0.05), hemoglobin (*p* < 0.05), and hematocrit (*p* < 0.05) concentrations compared to uninfected control hamsters. In a similar study in Brazil, investigators examined the effects of *L*. *interrogans* serovar *icterohaemorrhagiae* infection on hematological parameters in rats [37]. At 15 days post-infection, leptospirosis-infected rats had significantly lower hemoglobin (mean ± SD; 11.6 ± 0.46 versus 12.7 ± 0.46 g/dL; *p* < 0.05) and hematocrit (39% versus 43%; *p* < 0.05) concentrations compared to uninfected control rats. Findings from these studies suggest that *Leptospira* infection impairs host iron status.

A laboratory study using in vitro and in vivo methods was conducted to determine the role of heme oxygenase (*hemO*) in leptospirosis pathogenesis in golden hamsters [38]. Transposon mutagenesis was used to develop two strains of *L*. *interrogans*, serovar *Manilae hemO* mutant M484 and control *hemO* mutant M511 (i.e., the same mutant strain with a transposon insertion between 16S rRNA gene and LA2443 to control for attenuation during mutagenesis). Golden hamsters were inoculated with mutant M484 (*n* = 8), control *hemO* mutant M511 (*n* = 8), or wild-type parent *L*. *interrogans* serovar *Manilae* (*n* = 8). Hamsters inoculated with the *hemO* mutant M484 had a significantly higher survival rate compared to control *hemO* mutant M511 (83% versus 33%; *p* = 0.001) and wild-type parent *L*. *interrogans* serovar *Manilae* (83% versus 0%; *p* = 10^−6^). Findings suggest that although *hemO* is not required for acute disease onset, it may influence *Leptospira* virulence via heme degradation.

Several other in vivo studies have examined the effects of leptospirosis on hematological parameters, including hemoglobinemia, hemoglobinuria, and abnormal red blood cell size or shape [39–44]. In a study among golden hamsters, investigators inoculated weanling hamsters with *L*. *interrogans ballum* and collected blood after euthanization at two weeks to assess hematological status [43]. Blood smears from leptospirosis-infected hamsters had increased red blood cell destruction and hemoglobinemic nephrosis compared to uninfected hamsters. In a similar study, one experimental group of golden hamsters was irradiated and inoculated with *L*. *interrogans*; hematological parameters were assessed and compared to three control groups: irradiated, infected, or uninfected control hamsters [40]. Red blood cells from both of the infected groups had pitted spherocytes and increased hemoglobinemia compared to the biconcave disks of normal red blood cells from uninfected controls. Findings from these studies suggest that virulent strains of *Leptospira* adversely affect host erythrocytes, which may be due to heme acquisition for bacterial survival.

In a study among calves inoculated with *L*. *interrogans* serovar *hardjo*, calves with higher antibody levels had increased hemolytic anemia, compared to calves with lower antibody levels or uninfected cows [41]. Similarly, two other studies noted red blood cell disfiguration, hemoglobinemia, and hemoglobinuria in *L*. *interrogans*-infected calves [39] and hamsters [42], compared to uninfected animals.

In another study, 27 sheep were inoculated with different strains of *L*. *interrogans* to determine the effects on hematological parameters [44]. Anemia and hemoglobinuria were observed in two *L*. *interrogans pomona*-infected and one *L*. *interrogans canicola*-infected sheep of the 13 sheep infected with either of those strains, but not in the six sheep infected with the non-hemolytic serovar *hardjo* strain.

#### Human studies

Several observational studies have been conducted to examine the associations between iron status and leptospirosis in humans. A prospective cohort study was conducted in Sri Lanka among 201 patients admitted to a hospital with suspected leptospirosis and followed up for two weeks [45]. Leptospirosis was diagnosed based on WHO clinical criteria (i.e., acute febrile illness with headache and myalgia) and confirmed with the microscopic agglutination test (MAT titer >400 or a 4-fold rise between acute and convalescent samples). Severe leptospirosis was classified based on renal insufficiency (urine output <400 mL/day, creatinine >133 umol/L, and urea >25.5 mmol/L), jaundice (bilirubin >51.3 μmol/L), or death. Thrombocytopenia (platelets <150 x 10^9^/L) was observed in 56.8% and 73.8% of patients on the third and fifth days of follow-up, respectively. Patients with severe leptospirosis had significantly lower hemoglobin and hematocrit concentrations compared to patients with mild leptospirosis (3–10d; Hemoglobin [Hb]: 10–12 versus 12–14 g/dL; *p* < 0.0001).

In a prospective cohort study among patients with leptospirosis (*n* = 73) and dengue fever (*n* = 68) with two years of follow-up, thrombocytopenia (>100,000/mm^3^) was observed in 47% of leptospirosis patients (34/73) and in 77.7% of fatal leptospirosis cases (14/18) [6]. Thrombocytopenia was also more commonly reported in patients with leptospirosis compared to patients with dengue fever.

Several studies have noted a high prevalence of anemia and other hematological abnormalities in patients with leptospirosis. A cross-sectional study in Iran was conducted to examine hematological parameters among patients with leptospirosis (*n* = 74) [46]. Leptospirosis was diagnosed clinically (i.e., fever, headache, myalgia, and prostration) and confirmed via immunofluorescence antibody test for positive serology (antibody titer >1/100) or via a 4-fold or higher increase in anti-leptospire antibody titer between the first and second serum specimen (≥15-day interval). Hematological abnormalities were common: 87.3% of patients had thrombocytopenia (platelets <150,000 cells/mm^3^), 71.0% had hematuria (>3 red blood cell per high power field [RBC/HPF]), and 78.4% had anemia (male: Hb<13.0 g/dL, female: Hb<12.0 g/dL) at baseline.

In a case-control study in Australia among male patients infected with leptospirosis (*n* = 207), hemoglobin concentrations varied significantly by leptospirosis serovar (F = 2.67, *p* = 0.004) [47]. Patients infected with *L*. *interrogans Canicola* had significantly lower hemoglobin concentrations compared to all other serovars, including *L*. *interrogans Hardjo* (131.3 versus 146.3 g/L, *p* = 0.03), *Robinsoni* (131.3 versus 145.6 g/L, *p* = 0.02), *Tarassovi* (131.3 versus 145.3 g/L, *p* = 0.04), and *Zanoni* (131.3 versus 147.3 g/L, *p* = 0.02) serovars. Findings suggest that *L*. *interrogans Canicola* may be associated with the most severe hematological profile compared to other serovars, but the specific mechanisms are unknown.

In a case-control study, clinical records from leptospirosis patients hospitalized in Australia (*n* = 239) were compared to identify differentiating hematological markers between severe (*n* = 12) and uncomplicated (*n* = 227) leptospirosis [48]. Patients with severe leptospirosis (i.e., respiratory distress, dyspnea, hemoptysis, diffuse alveolar hemorrhage, and/or acute liver or renal failure) had significantly lower mean hemoglobin concentrations (mean ± SD; severe: 122.3 ± 0.66 versus uncomplicated: 145.3 ± 0.90 g/L; *p* = 0.005), platelet counts (mean ± SD; severe: 109.8 ± 20.2 versus uncomplicated: 162.4 ± 3.8 x10^9^/L; *p* = 0.03), hematocrit (mean ± SD; severe: 0.36 ± 0.09 versus uncomplicated: 0.43 ± 0.003; *p* = 0.003), and erythrocyte counts (mean ± SD; severe: 4.1 ± 0.02 versus uncomplicated: 4.8 ± 0.03 x10^12^/L; *p* = 0.01), compared to uncomplicated leptospirosis cases.

A retrospective cohort study in France was conducted to evaluate hematological parameters among patients with confirmed leptospirosis (*n* = 34; clinical criteria: antibody titer >1:400 and positive MAT titer >1:100) [49]. Anemia (*n* = 4; men: Hb<12.0 g/dL, women: Hb<11.0 g/dL), hematuria (*n* = 6; positive urine strip test), and thrombocytopenia (*n* = 6; platelets <150,000 cells/mm^3^) were the most common hematological abnormalities.

A case-control study was conducted in Puerto Rico to compare hematological parameters among patients with leptospirosis (*n* = 42) to patients with dengue fever (*n* = 84) [50]. Hematological abnormalities were common among patients with leptospirosis, including thrombocytopenia (85%; platelets <100,000 cells/mm^3^), hematuria (71%), and anemia (62%; male: Hb<13.8 g/dL, female: Hb<12.1 g/dL). The odds of anemia (*p* < 0.01) and hematuria (*p* < 0.01) were significantly higher among patients with leptospirosis compared to patients with dengue fever.

A retrospective cohort study was conducted in Moldova to examine clinical presentations of 58 patients with leptospirosis (4-fold increase in initial ELISA titer or ≥400 MAT titer) with acute renal failure (serum creatinine >150 mmol/L) over five years of follow-up [51]. A total of 72.4% of patients exhibited hemolytic anemia (i.e., polychromatophilia, high unconjugated bilirubin, raised lactate dehydrogenase levels, and reticulocytosis).

In a prospective cohort study in Korea, patients with acute febrile illnesses were hospitalized and tested for leptospirosis [52]. Leptospirosis was confirmed with the microscopic agglutination test (i.e., a titer of agglutination of >50% in a single specimen or a 4-fold increase in titers between acute and convalescent samples). Of 93 confirmed leptospirosis cases, 37% of patients had moderate to severe anemia.

Overall, laboratory studies have demonstrated that leptospirosis impairs iron status. In vitro studies identified heme oxygenase and chemotaxis as hemolytic mechanisms for pathogenic leptospiral heme acquisition and confirmed that iron is required for leptospire growth and survival. Animal studies noted lysed erythrocytes, hemoglobinemia, and hemoglobinuria when infected with leptospirosis compared to uninfected animals. Human studies also demonstrated an association between leptospirosis and impaired host hematological status, including lower hemoglobin and hematocrit concentrations, anemia, and thrombocytopenia.

### Calcium

Calcium (Ca^2+^) is an important second messenger molecule in immune function. Elevation in cytosolic free calcium triggers immune cell activation and operation, including B- and T-cell lymphocytes and macrophages [53]. During inflammatory tyrosine kinase cascades, Ca^2+^ efflux activates plasma membrane Ca^2+^ channels and enables store-operated Ca^2+^ entry (SOCE) [54,55]. SOCE is required for a variety of immune functions, including regulatory T-cell development and function, CD4 T-cell cytokine production, cytotoxic lymphocyte (i.e., CD8 T-cells, NK cells) cytokine response, B-cell differentiation, macrophage nitric oxide production, and secretion of antimicrobial and pro-inflammatory factors [56,57]. Experimental chelation studies have demonstrated calcium’s role in mast cell activation; blocking Ca^2+^ mobilization also prevented mast cell degranulation and release of inflammatory mediators [54,58].

#### In vitro studies

Several in vitro studies have been conducted to examine the role of calcium in *Leptospira* infection. In an in vitro study in human THP-1 and mouse macrophages, macrophages were infected with *L*. *interrogans*, and changes in Ca^2+^ concentrations and risk of cell death were compared in infected versus uninfected macrophages [59]. *Leptospira*-infected macrophages had significantly elevated Ca^2+^ concentrations, apoptosis, and necrosis compared to uninfected cells (*p* < 0.05). Similarly, addition of Ca^2+^ chelator EGTA significantly reduced Ca^2+^ elevations (as detected by fluorescence) in *Leptospira*-infected macrophages compared to uninfected macrophages. Findings suggest that Ca^2+^ may influence macrophage survival and host immune response in leptospirosis.

Several in vitro studies have examined the associations between calcium and major lipoprotein 32 (LipL32) of pathogenic *Leptospira*. In an in vitro study, LipL32 mutants were constructed to determine the role of the Ca^2+^ binding cluster of the protein [60]. Results indicated that Ca^2+^ binding was essential for regulation of LipL32 interaction with toll-like receptor-2 (TLR2), suggesting a potential role for calcium in immune response to *Leptospira* infection. In another study, LipL32 was isolated and transfected into *E*. *coli* cells in order to assess LipL32 binding affinity to Ca^2+^ and human fibronectin (F30) [61]. Analysis of circular dichroism (CD) spectra demonstrated that Ca^2+^ promotes LipL3 binding to fibronectin; binding affinity for F30 was stronger for Ca^2+^-bound LipL32 than for Ca^2+^-free LipL32 (K_d_ mean ± SD: 0.29 ± 0.29 versus 1.15 ± 0.06 μm; R^2^ = 0.99, *p* < 0.0001). These findings provide support for the hypothesis that calcium modulates pathogen outer membrane protein binding via LipL32 binding to fibronectin of the host cell and may influence host immune response.

In contrast, another in vitro study noted contradictory findings regarding the interactions between Ca^2+^ and LipL32 and their associations with *Leptospira* virulence. LipL32 was isolated and transfected into *E*. *coli* cells to generate mutants in order to assess protein affinity for Ca^2+^, human plasminogen, and fibronectin [62]. LipL32 mutants bound to plasminogen demonstrated similar affinities in conditions with and without Ca^2+^ present, suggesting that calcium is not required for LipL32 binding to host extracellular proteins.

There is conflicting evidence regarding the role of LipL32 during *Leptospira* infection. Although several in vitro studies have noted the importance of *Leptospira* outermembrane proteins (OMPs) during infection and the relative abundance of LipL32 [60,61,63–65], recent studies have suggested that LipL32 may instead be a subsurface lipoprotein [66] with no association to virulence [62,67]. For example, in one study, transposon mutagenesis was used to construct an *L*. *interrogans* LipL32 mutant and evaluate the protein’s role as a virulence factor [67]. The LipL32 mutant exhibited similarly efficient colonization of renal tubules in rats as the wild-type strain, suggesting that LipL32 is not required in infection. However, another study was conducted to investigate the location of LipL32 on *Leptospira* using surface proteolysis and immunofluorescence assays [66]. In contrast to earlier studies, this analysis established LipL32 as a subsurface protein connected to the inner layer of the lipid bilayer, indicating why previous studies localized LipL32 as an OMP.

Overall, in vitro studies suggest that calcium may influence host immune response to leptospirosis through two potential mechanisms: elevated extracellular calcium promotes host macrophage death, while calcium modulates binding via LipL32. However, conflicting evidence about the role of LipL32 during infection renders its potential association with calcium uncertain. Further research is needed to examine the role of calcium in the context of leptospirosis in in vivo animal and human studies.

### Zinc, Magnesium, and Other Trace Minerals

Trace minerals are essential for innate and adaptive immunity. For example, zinc is required for immune cell proliferation, dismutase activity against oxidative stress, and cytokine release [15]. In previous studies, zinc deficiency has been associated with decreased neutrophil phagocytosis, natural killer (NK) cell cytotoxicity, and T-cell function [68], while zinc supplementation improved innate immunity, macrophage phagocytosis, NK cell activity, CD8^+^ T-cell proliferation, and antibody response [14,15].

Magnesium has also been investigated in the context of immunity and infectious diseases. Magnesium is required in several organ systems, including renal function, heart contractibility, and neurotransmitter function. Renal dysfunction is a common clinical manifestation of leptospirosis; damage to the thick ascending limb of the Loop of Henle, the primary site of magnesium reabsorption, may adversely affect magnesium reabsorption and renal function [69].

Selenium deficiency has been classically associated with Keshan disease, a congestive cardiomyopathy named for the province in northeast China where it first presented [70–73]. Keshan disease is caused by a combination of dietary selenium deficiency and the presence of a mutated Coxsackievirus strain. Selenium deficiency results in increased oxidative stress due to disruption of the active site of glutathione peroxidase, resulting in myocardial inflammation and cardiomyopathy [73]. Myocarditis can result from a variety of viral and bacterial infections, and *Leptospira* has been cited as a spirochetal cause of myocarditis [74]. Although no studies to date have been conducted to investigate the role of selenium in leptospirosis in humans, the connection between selenium deficiency and myocarditis in infectious diseases represents a potential area for future research.

#### In vitro studies

An in vitro study was conducted to examine *L*. *interrogans* serovar *Icterohaemorrhagiae* outermembrane gelatinase activity dependence on metal ions, since gelatinase was hypothesized to be involved in leptospirosis host invasion [75]. The addition of metal chelators (100 mM of ethylenediaminetetraacetic acid [EDTA] and ethylene glycol tetraacetic acid [EGTA]) decreased gelatinase activity by 60% to 70%, while addition of metal ions (100 μm), Cu^2+^, Mn^2+^, Mg^2+^, or Zn^2+^ increased gelatinase activity (*p* < 0.05). Iron was the only metal that inhibited gelatinase activity (40%; *p* < 0.05). Findings suggested that pathogenic *Leptospira* strains exhibit substantial gelatinase activity compared to nonpathogenic strains and identified a potential mechanism for trace metals in leptospirosis pathogenesis.

Several in vitro studies were conducted to determine the effects of trace elements on *Leptospira* spp growth. In one study, the effects of Mn^2+^ or Fe^2+^ on leptospire growth were examined in wild-type serovars compared to LEPBla2866 mutant *Leptospira* in which ATP-binding cassette (ABC) ATPase was inactivated [76]. The wild-type *Leptospira* grew 100% in media with either Mn^2+^ or Fe^2+^, while the LEPBla2866 mutant had a 50% reduction in growth in the medium containing Mn^2+^ (*p* = 0.05). Findings suggest that a functional ATPase is needed for optimal leptospire growth in the presence of trace metals Mn^2+^ or Fe^2+^.

Another study was conducted to examine the effects of various mineral concentrations on leptospire growth; lower zinc and cobalt concentrations (0.2 x 10^−6^ g and 0.2 x 10^−9^ g, respectively) stimulated leptospiral growth, while higher concentrations of these minerals inhibited growth and resulted in toxicity [77,78]. However, in another experiment on trace minerals and leptospire growth in ten different strains, zinc nitrate significantly increased growth in all strains; the optical density of *Leptospira naam naam* increased from 0.38 to 0.49 (log(OD)/hour; *t* = 15.07, *p* = 0.001) [79]. Findings from these in vitro studies suggest that zinc may influence leptospiral growth.

In a study of trace minerals and leptospire growth in *Leptospira pomona*, media supplemented with calcium and magnesium stimulated leptospire growth, while magnesium deficiency (<5 x 10^−7^ M) inhibited growth [80]. Metal inhibition by chelator EDTA demonstrated that 10^−4^ M concentrations of divalent metal cations was required for *L*. *pomona* growth [80]. In an in vitro study of minerals and leptospire growth in *Leptospira canicola*, different media were prepared without trace minerals (Ca^2+^, Ba^2+^, Sr^2+^, Mg^2+^, or Co^2+^); only Ca^2+^ was required for growth, and low magnesium concentrations inhibited but did not completely prevent growth [81].

#### Animal studies

In an in vivo study, hamsters were inoculated with *L*. *interrogans* serovar *Copenhageni*, and serum sodium, potassium, and magnesium concentrations were measured and compared to healthy control hamsters [82]. Serum potassium (*p* < 0.05) and magnesium (day 4; *p* < 0.05) concentrations were significantly higher in leptospirosis-infected hamsters compared to uninfected hamsters.

In an observational study, 225 sea lions were referred to marine mammal rehabilitation centers for leptospirosis testing [83]. Blood samples were collected upon admission to evaluate associations of *L*. *interrogans* serovar *Pomona* infection with serum calcium, phosphorus, and potassium concentrations [83]. A total of 86 sea lions were diagnosed with *L*. *interrogans* infection (MAT titer >1:3,200). Sea lions with higher serum phosphorus concentrations (>7.0 mg/dL) had 6.8 times greater odds of being infected with *L*. *interrogans* serovar *Pomona* compared to those with lower phosphorus levels (≤7.0 mg/dL; odds ratio [OR]: 6.8, 95% confidence interval [95% CI]: 2.4–20.3, *p* < 0.05). Similarly, sea lions with higher calcium concentrations (>8.9 mg/dL) had 16.8 times greater odds of being infected with *L*. *interrogans* serovar *Pomona* compared to those with lower calcium levels (≤8.9 mg/dL; OR: 16.8, 95% CI: 4.3–70.0, *p* < 0.05). However, there were no significant differences in the odds of *L*. *interrogans* infection for elevated potassium (>4.2 mEq/L; OR: 2.3, 95% CI: 0.8–6.1, *p* > 0.05) or sodium (>151 mEq/L; OR: 1.7, 95% CI: 0.7–4.3, *p* > 0.05) concentrations.

In a study in New Zealand among grivet monkeys (*n* = 13), serum zinc and iron concentrations were compared between monkeys inoculated with either *L*. *interrogans balcanica* or *L*. *interrogans tarassovi* [84]. There were no significant differences in serum zinc or iron concentrations.

A study was conducted among golden hamsters to determine leptospire micronutrient requirements for survival within the host [85]. Hamsters were inoculated with *L*. *pomona* or *L*. *canicola moulton* and euthanized upon presentation of hemorrhagic renal or hepatic lesions. Virulent leptospires were then obtained from host tissue and grown in vitro; leptospire growth was determined by cell density and cell count. *L*. *canicola* were cloned and administered to hamsters in order to develop high and low virulence strains from the same serovar. Media supplemented with Mg^2+^ (2.0 mM) stimulated growth of virulent *L*. *canicola* strains but did not alter the growth of avirulent strains.

Another study was conducted in golden hamsters to examine the effects of selenium on leptospire growth [86]. Selenium test compounds were added to seven different *Leptospira* strains in media, and hamsters were inoculated and euthanized 30 days after infection. Selenourea (5 mg/kg), a synthetic organic compound of selenium, inhibited leptospire growth for all strains except *L*. *canicola* and *L*. *icterohaemorrhagiae*; virulent strains were more resistant than non-virulent strains.

#### Human studies

Two observational studies have been conducted to examine the association between leptospirosis and magnesium status among patients with leptospirosis. In a retrospective cohort study in Australia among patients with severe leptospirosis (*n* = 15; MAT titer ≥400 or 4-fold increase in titers between onset and convalescence), serum magnesium concentrations were measured over a 10-day period [87]. Hypomagnesemia (Mg^2+^ <0.70 mmol/L) was observed in 93% of patients (*n* = 14/15). Hypomagnesemia may be the result of renal failure in leptospirosis-infected patients, at the thick ascending limb of the Loop of Henle, the main site of magnesium reabsorption.

In a prospective cohort study in Thailand among patients with leptospirosis (*n* = 20; MAT titer ≥4-fold or ≥1:200), renal function and urine and serum magnesium, calcium, and creatinine concentrations were assessed [69]. At admission, ten patients exhibited hypomagnesemia (Mg^2+^ <0.7 mmol/L), 15 patients had renal magnesium wasting, and five patients had hypocalcemia (Ca^2+^ <2.0 mmol/L). After two weeks, fractional magnesium excretion was significantly greater in leptospirosis patients with acute renal failure compared to those without renal failure (10.1 versus 3.1%; *p* < 0.01). Findings suggest that lower serum magnesium concentrations are associated with renal failure in patients with leptospirosis.

There is relatively limited evidence regarding the role of trace elements in leptospirosis. In vitro studies have established minimum micronutrient requirements of divalent cations for leptospirosis survival. However, the functions of trace elements in leptospirosis pathogenesis or mechanisms involved have not been established. Findings from two cohort studies suggest that hypomagnesemia is associated with leptospirosis and renal failure, a common clinical complication of leptospirosis and risk factor for mortality.

### B-Vitamins

B-vitamins, including vitamin B_12_ and folate, have important roles in immune function. Vitamin B_12_ deficiency has been associated with impaired NK cell function [88] and reduced lymphocyte and CD8^+^ T-cell counts [89]. Folate deficiency has also been associated with reduced T-cell proliferation in response to mitogen activation, lowered resistance to infections, and increased CD4:CD8 T-cell ratios [88,90]. Additionally, vitamin B_6_ deficiency has been associated with impaired lymphocyte proliferation, antibody production, and NK cell and T-cell cytotoxicity [91].

#### In vitro studies

Few studies to date have been conducted to examine the role of B-vitamins in leptospirosis. Several in vitro studies have demonstrated that vitamin B_12_ and thiamine are essential for leptospire growth and survival through experiments suspending leptospires in cultures with titrated ranges of vitamin concentrations [92–96]. In an in vitro study examining the role of B-vitamins in leptospirosis, titrated concentrations of riboflavin, pyridoxine, thiamine, biotin, nicotinic acid, and folic acid increased the optical density of leptospire cultures, an indicator of increased growth [97].

#### Animal studies

A study was conducted among hamsters to determine the effects of vitamin B_12_ on leptospire growth; investigators suspended leptospires in culture with a range of vitamin B_12_ concentrations and inoculated hamsters with solutions of vitamin B_12_ and leptospires [98]. Vitamin B_12_ was associated with increased leptospire growth, and the minimum concentrations of vitamin B_12_ required for growth were determined to be 0.16 x10^-4^ to 0.16 x10^-9^ μg/mL [98]. Another study in hamsters also demonstrated that vitamin B_12_ and thiamine were essential for leptospiral growth [99].

In vitro and in vivo studies have established minimum B-vitamin requirements for leptospiral growth and survival. However, the role of B-vitamins in host defense or leptospirosis pathogenesis or specific mechanisms has not been established.

## Discussion

The evidence of the role of micronutrients in leptospirosis is relatively limited and heterogeneous. Evidence from laboratory studies suggests that micronutrients such as iron, calcium, and magnesium may be associated with leptospirosis. Few prospective studies and no randomized trials have been conducted to date to determine the role of micronutrients in *Leptospira* infection. Laboratory studies demonstrated that iron is required for leptospire growth and survival, and findings from animal and human studies to date have noted an association between leptospirosis and impaired host hematological status. Additionally, calcium influences host macrophage apoptosis and binding to a major *Leptospira* lipoprotein, LipL32, suggesting that elevated calcium may indicate leptospirosis disease progression. Hypomagnesemia has been noted during leptospirosis in two observational cohort studies, which may be related to renal failure in patients with leptospirosis. Comparatively little research has been conducted to examine the role of other micronutrients in *Leptospira* infection and pathogenesis.

Research to date on micronutrients and leptospirosis has primarily focused on iron and has identified its potential in leptospire infection and pathogenesis. Laboratory studies demonstrated that iron is required for leptospire growth and survival: leptospires lyse host erythrocytes to obtain iron, and iron limitation suppresses leptospire growth. Observational human studies have noted an association between leptospirosis and impaired hematological status, including higher prevalence of anemia and lower hemoglobin and hematocrit concentrations. However, further research is needed to inform screening and appropriate clinical management in at-risk populations.

Limited research to date has been conducted on the role of calcium in leptospirosis. However, preliminary findings from in vitro studies suggest that calcium is required for *Leptospira* infection. Several in vitro studies have examined the associations between calcium and major lipoprotein LipL32 of pathogenic leptospires, a potential virulence factor, and suggest that calcium modulates leptospire binding to host fibronectin and subsequent leptospirosis pathogenesis [60–62]. Additionally, calcium may influence macrophage survival and host immune response during *Leptospira* infection. However, in vivo and human studies are needed to determine the role of calcium in host defense and immune response in leptospirosis.

Hypomagnesemia has been associated with *Leptospira* infection and disease severity in both in vitro and observational human studies. Hypomagnesemia often appears in hospitalized patients and has been identified as a risk factor for renal failure in cohort studies among leptospirosis-infected patients [69,87], while sufficient magnesium status and magnesium supplementation may reduce the risk of renal failure and associated mortality in patients with leptospirosis [100]. Renal dysfunction is a common clinical presentation in patients with leptospirosis and has been associated with increased risk of mortality.

The temporal association between hypomagnesemia and renal failure is not clear: magnesium is required for renal function, and hypomagnesemia is a risk factor for renal failure; however, renal dysfunction (and damage to the thick ascending limb of the Loop of Henle, the primary site of magnesium reabsorption) also adversely affects magnesium reabsorption and status [69]. Prospective studies with larger sample sizes and intervention trials are needed to establish the role of magnesium in *Leptospira* infection and severity. Additionally, although no studies to date have been conducted on the role of selenium in leptospirosis, the connection between selenium deficiency and myocarditis in other infectious diseases (e.g., Keshan disease) represents a potential area for future research.

This review has several limitations. The majority of studies to date have been small mechanistic in vitro studies, and only 11 observational studies to date have been conducted in human populations. Most studies to date have focused on iron (30 of 56), calcium, and magnesium; there are relatively few studies examining the role of other micronutrients in leptospirosis. Micronutrients are also modulated by other factors, including inflammation, clinical disease severity, and other nutrients, which were not measured or controlled for in these studies. Furthermore, no randomized trials have been conducted to date to determine the effects of micronutrients on *Leptospira* infection and pathogenesis. The role of micronutrients in leptospirosis risk and severity needs to be elucidated in larger prospective human studies and intervention trials to inform public health interventions.

In summary, studies have indicated that higher iron and calcium concentrations and hypomagnesemia may be associated with *Leptospira* infection and highlighted a potential role of calcium during initial pathogenesis. However, few prospective studies and no randomized trials have been conducted to date to examine the potential role of micronutrients in *Leptospira* infection. The limited literature in this area constrains our ability to make specific recommendations; however, the role of iron, calcium, and magnesium in leptospirosis represent important areas for future research. In the context of active surveillance, water sanitation, hygiene programs, clinical care, and treatment programs, micronutrient supplementation could represent an important intervention to reduce the burden of leptospirosis and inform public health interventions in at-risk populations.

**Key Learning Points**

- Leptospirosis cases are often misdiagnosed and underreported due to similar clinical presentation as other acute febrile illnesses.
- Micronutrient supplementation could represent an important intervention to reduce the burden of leptospirosis and inform public health interventions in at-risk populations.
- Few prospective studies and no randomized trials have been conducted to date to examine the potential role of micronutrients in *Leptospira* infection.
- Higher iron and calcium concentrations and hypomagnesemia may be associated with *Leptospira* infection.
- Gaps in research suggest a need for increased leptospirosis surveillance and prospective studies in at-risk human populations.

**Five Key Papers in the Field**

- Scrimshaw NS. Nutritional status and infectious disease. Ill Med J 1962;122:467–475.
- De Silva NL, Niloofa M, Fernando N, Karunanayake L, Rodrigo C, et al. Changes in full blood count parameters in leptospirosis: a prospective study. Int Arch Med 2014;7:31.
- Craig SB, Graham GC, Burns MA, Dohnt MF, Smythe LD, et al. Haematological and clinical-chemistry markers in patients presenting with leptospirosis: a comparison of the findings from uncomplicated cases with those seen in the severe disease. Ann Trop Med Parasitol 2009;103:333–341.
- Lo YY, Hsu SH, Ko YC, Hung CC, Chang MY, et al. Essential calcium-binding cluster of Leptospira LipL32 protein for inflammatory responses through the Toll-like receptor 2 pathway. J Biol Chem 2013;288:12335–12344.
- Khositseth S, Sudjaritjan N, Tananchai P, Ong-ajyuth S, Sitprija V, et al. Renal magnesium wasting and tubular dysfunction in leptospirosis. Nephrol Dial Transplant 2008;23:952–958.

## Supporting Information

S1 Table Iron. Iron: Evidence from in vitro, animal, and human studies of the association between iron and *Leptospira* infection.
(DOCX)

S2 Table Calcium. Calcium: Evidence from in vitro laboratory studies of the association between calcium and *Leptospira* infection.
(DOCX)

S3 Table Trace minerals. Trace minerals: Evidence from in vitro, animal, and human studies of the association between trace minerals and *Leptospira* infection.
(DOCX)

S4 Table B-vitamins. B-vitamins: Evidence from in vitro and animal laboratory studies of the association between B-vitamins and *Leptospira* infection.
(DOCX)
