# Supplementary material for: Micronutrients and Leptospirosis: A Review of the Current Evidence
Source: PLoS Negl Trop Dis. 2016 Jul 7;10(7):e0004652. doi: 10.1371/journal.pntd.0004652 (PMC4936698; doi:10.1371/journal.pntd.0004652)
Supplement: S1 Table — Iron: Evidence from in vitro, animal, and human studies of the association between iron and Leptospira infection. (DOCX) [file pntd.0004652.s001.docx]

**S1 Table. Iron**

| **Authors** | | **Sample (N)** | **Methods** | **Definition of Leptospirosis** | **Definition of Micronutrient** | **Main Findings** | |
| --- | --- | --- | --- | --- | --- | --- | --- |
| ***Laboratory*** | | | | | | | |
| [[31](#_ENREF_31)] | | 1 x10^9^ leptospires/mL | Transposon insertion inactivated *La4131* metalloprotease to evaluate the effects of iron overload on cell growth | *L. interrogans* serovar *Lai* strain LL55601, parent strain L521, *La4131* transposon mutant strain L522 | Iron overload: supplement EMJH with 360 μM FeSO_4_. Differential expression: genes up/down-regulated at least 2-fold | No difference in growth rate between parent L521 and mutant L522 in EMJH*. La4131* inactivation reduced transcript levels 8-fold compared to parent (P<0.01) and altered gene expression of 13 genes in EMJH + iron | |
| [[28](#_ENREF_28)] | | 2.5x10^8^-6.5x10^8^ leptospires/mL | Microarray analysis evaluated transcriptional response of *L. interrogans* to iron limitation and *la1857* mutation | *L. interrogans* serotype *Manilae*, mutant Fur homolog *la1857* (regulates iron uptake and storage) | Iron limitation: EMJH with 40 μM 2,2’-dipyridyl. Differential expression: genes up/down-regulated at least 2-fold | *La1857* mutation did not significantly affect expression of iron-responsive genes. Under iron limitation, 43 genes were up-regulated and 49 genes down-regulated. 16.7% of down-regulated genes affected cell division and cell cycle control vs. 7.1% across the genome (p=0.012, R^2^=0.7287) | |
| [[29](#_ENREF_29)] | | 1x10^8^ leptospires/mL | Immunoblot analysis evaluated gene expression levels under iron limitation for *L. interrogans* cultures | *L. interrogans* serotype *Copenhageni* | Iron limitation: EMJH with 0.4 mM 2,2-dipyridyl | Iron limitation up-regulated 6 proteins associated with virulence. 17 genes in protein synthesis for metabolism and energy production down-regulated (p<0.05) | |
| [[30](#_ENREF_30)] | | 2.5x10^8^- 7.5x10^8^ leptospires/mL | Transposon mutagenesis created mutant heme oxygenase gene *HemO* (LB186); ability to use hemoglobin (Hb) was evaluated | *L. interrogans* serotype *Manilae* | Standard EMJH + 36 μM FeSO_4_, EMJH without iron, EMJH + 0.3 μM rabbit Hb solution, and/or 36 μM FeSO_4_ | Mutant lacking *HemO* exhibited normal growth in EMJH similar to wild type (p<0.05). Parent and mutant unable to grow in EMJH lacking iron | |
| [[27](#_ENREF_27)] | | n/a | Reverse-transcription PCR evaluated gene expression of cells grown in normal and in iron-limited conditions, and with different iron sources | *L. biflexa* serotype *Patoc* strain Patoc1 and  *L. interrogans* serotype *Lai* strain Lai | Limited iron: omit iron sulfate from EMJH or EMJH with 50 μM 2,2-dipyridyl. Iron sources for growth tests: EMJH + 100 μM iron sulfate, 100 uM iron chloride, 100 μM iron, 10 μM hemin | *L. biflexa* and *L. interrogans* use iron chloride, iron sulfate, iron citrate hemin/Hb as an iron source but not lactoferrin. In iron limited conditions, fur2, fur3, and fur4 decreased transcription 10-fold, but there were no changes in fur1 expression | |
| [[24](#_ENREF_24)] | | 5x10^8^ bacteria/mL | Random transposon mutagenesis of *L. biflexa* with transposon *Himar1* to evaluate effects of iron limitation on growth | *L. biflexa* serotype Patoc strain Patoc I | Limited iron: FeSO_4_ omitted from EMJH or EMJH with 0.1 mM 2,2-dipyridyl | Limited iron inhibited both wild-type and mutant growth. 10 μM hemin, 10 μM deferoxamine, and 100 μM ferric dicitrate restored growth for iron-limited *leptospira* | |
| [[23](#_ENREF_23)] | | *E. coli* strains grown in LB medium | *Leptospira* heme genes transformed into *E. coli* cultures to evaluate effect of heme limitation on growth | *L. interrogans* serotype *icterohaemorrhagiae* and *L. biflexa* serotype *patoc* | EMJH + hemin or Hb to final concentration of 0-100 μM (0, 1, 5, 10, 20, 50 and 100 μM | *L. biflexa* wild strain showed no difference in growth rate from 0-100 μM hemin. *L. biflexa* mutant had inhibited growth in EMJH with limited hemin; 5 μM hemin supplement restored mutant growth | |
| [[32](#_ENREF_32)] | | Leptospire cultures, bovine Hb | *Leptospira* strains assessed for chemotaxis to Hb and association with virulence | 4 virulent and 14 avirulent *L. interrogans* strains | Chemotaxis toward Hb (21, 2.1, and 0.21 μg Hb) | Avirulent strains of *L. interrogans* and saprophytic strains of *L. biflexa* did not exhibit significant chemotaxis toward Hb. Virulent strains exhibited significant chemotaxis toward Hb (p<0.01) | |
| [[22](#_ENREF_22)] | | 1x10^9^ leptospires/mL | Outer membrane protein activity compared in two flasks with iron chelators to control with sufficient iron | *L. alstoni* serotype *grippotyphosa, L. borgpetersenii* serotype *hardjo, L. interrogans* serotypes *autumnalis, Bratislava, canicola, icterohaemorrhagiae*, and *pomona* | Iron-limitation 200 μM of 2,2’-dipyridyl or EDDA (Ethylenediamine diacetic acid)  Sufficient iron: 200 μM ferric chloride | Serotypes grown in low iron to OD_600_ (optical density) of 0.08: growth rate reduced within 12 hours with 2,2’-dipyridyl growth and within 24 hours with EDDA. EDDA culture OD_600_ = 0.124, dipyridyl culture OD_600_ = 0.083, and low iron control OD_600_ = 0.168. Outer membrane protein expression was not dependent on iron availability | |
| [[88](#_ENREF_88)] | | Human, rabbit, and sheep blood erythrocyte suspensions | Evaluate leptospiral hemolysis in human and sheep blood erythrocyte suspensions and effect of CaCl_2_ addition on hemolysis | Leptospiral virulent and saprophytic strains | n/a | Hemolysis activated by CaCl_2_ addition in rabbit and human erythrocytes | |
| [[25](#_ENREF_25)] | | Sheep cells | Evaluate effect of iron concentrations on leptospiral growth | *L. icterohaemorrhagiae, L. Pomona, L. canicola* | Korthof medium, 250–350 μg Fe per 100 mL (ferrous ammonium sulphate) | Addition of Hb and iron stimulated growth | |
| [[31](#_ENREF_31)] | | 1 x10^9^ leptospires/mL | Transposon insertion inactivated *La4131* metalloprotease to evaluate the effects of iron overload on cell growth | *L. interrogans* serovar *Lai* strain LL55601, parent strain L521, *La4131* transposon mutant strain L522 | Iron overload: supplement EMJH with 360 μM FeSO_4_. Differential expression: genes up/down-regulated at least 2-fold | No difference in growth rate between parent L521 and mutant L522 in EMJH*. La4131* inactivation reduced transcript levels 8-fold compared to parent (P<0.01) and altered gene expression of 13 genes in EMJH + iron | |
| ***Animal Studies*** | | | | | | | |
| [[33](#_ENREF_33)] | 28 hamsters | | Serum ferritin, transferrin, and bone marrow status evaluated from 2 groups of negative controls and 2 experimental groups of infected animals | 2 groups of hamsters were infected with *L. interrogans* serotype *Pomona* | Iron status assessed by serum iron, transferrin, ferritin, iron binding capacity, transferring saturation index | | Significantly higher serum iron (p<0.01), ferritin (p<0.01), and hepcidin (p<0.01) in infected animals compared to controls. Significantly lower transferrin (p<0.05), Hb, and hematocrit (p<0.01) in infected animals compared to controls. |
| [[34](#_ENREF_34)] | 24 rats; negative control  Group A;  Group B inoculated | | Adenosine deaminase activity and hematological parameters were compared between infected vs. control rats | Rats infected with *L. interrogans* serotype *icterohaemorrhagiae*, 2x10^8^ leptospires per rat | Hb and hematocrit | | Hb was significantly lower in *Leptospira*-infected rats compared to control rats on day 15 (11.6 ± 0.46 g/dL vs. 12.7 ±0.46 g/dL; p<0.05). Hematocrit (%) was significantly lower in infected group on Day 15 compared to un-infected (p<0.05) |
| [[35](#_ENREF_35)] | 8 hamsters injected with 10^3^ leptospires in 100 μL EMJH medium | | Hamsters inoculated with *HemO* mutant M484 (via transposon mutagenesis) to determine whether *HemO* required for pathogenesis. Negative controls inoculated with 100 μL EMJH. | *L. interrogans* serotype *Manilae, L. interrogans* serotype *Manilae* *HemO* mutant M484 | n/a | | *L. interrogans* requires heme oxygenase for disease pathogenesis. Hamsters infected with *HemO* mutant (M484) showed significantly different survival rate from control mutants and wild type (83% vs. 33% vs. 0% survival, p=0.001, p=10^-6^). |
| [[40](#_ENREF_40)] | Weanling golden hamsters inoculated | | Hematological parameters measured after euthanasia of inoculated hamsters | *L. interrogans* serotype ballum or *L. interrogans* serotype *Pomona (*1x10^7^ leptospires) | n/a | | *L. interrogans* serotype ballum associated with RBC (red blood cell) destruction and hemoglobinemic nephrosis |
| [[37](#_ENREF_37)] | 52 golden hamsters:  12 untreated controls, 12 irradiated controls, 14 infected. 14 irradiated and infected | | Blood samples of irradiated, *L. interrogans*-infected hamsters compared to infected controls, irradiated controls, and untreated controls to assess effects of impaired immunity on hemoglobinemia | Hamsters infected with 10^7^ organisms per mL of *L. interrogans* serotype *ballum* | Qualitative observations of RBC size/shape | | RBCs from untreated controls were biconcave disks. RBCs from both infected groups were pitted spherocytes and associated with increased hemoglobinemia |
| [[38](#_ENREF_38)] | 265 cows | | Blood and urine samples collected from cattle herd tested for leptospirosis | *L. interrogans* serotype *hardjo* confirmed by  MAT (tenfold serum dilution scheme, reaction criterion of 50% agglutination of antigen) for positivity. Positive: 10^-2^ or higher reaction | n/a | | Hemolytic anemia associated *L. interrogans* infection (titer > 10^-4^ antibodies) |
| [[36](#_ENREF_36)] | 10 calves | | Hematological parameters inoculated calves were measured | 1x10^7^ leptospires, *L. interrogans* serotype *pomona* | n/a | | Hemoglobinemia and hemoglobinuria observed in 3 infected calves. RBCs from infected animals exhibited disfigured shape |
| [[39](#_ENREF_39)] | Weanling hamsters | | RBC destruction qualitatively assessed in hemoglobinemic hamsters inoculated with *L. interrogans* | 1x10^7^ *L. interrogans* serotypes *Pomona* and *ballum* | n/a | | *Ballum*-infected hamsters exhibited hemoglobinemia and abnormal RBCs; *Pomona*-infected hamsters had normal Hb concentrations, were not hemoglobinemic, and had normal RBCs |
| [[41](#_ENREF_41)] | 27 sheep: 3 infected groups and group 4 uninfected controls | | Sheep were inoculated with *L. interrogans.* Blood samples collected at time of infection and for 12 days after to measure anemia and hemoglobinuria | *L. interrogans*; hemolysin-producing strains of serotypes *Pomona* and *canicola* and nonhemolytic strain of serotype *hardjo* | n/a | | Two *Pomona*-infected and one *canicola*-infected sheep had anemia and hemoglobinuria |
| **Human Studies** | | | | | | | |
| **Authors** | **Sample (N)** | | **Methods** | **Definition of Leptospirosis** | **Definition of Micronutrient** | | **Main Findings** |
| [[42](#_ENREF_42)] | 201 patients hospitalized with leptospirosis | | Prospective cohort study; hematological parameters measured at 14 days | MAT titer >400 or 4-fold rise between acute and convalescent samples. Severe leptospirosis classified: renal insufficiency (urine output <400 mL/day, creatinine >133 μM>1/L, urea >25.5 mmol/L); jaundice (bilirubin >51.3 μM/L), prolonged hospital stay (>10 days) | Thrombocytopenia: <150x10^9^ platelets/L | | Thrombocytopenia seen in 56.76% of patients on day 3 and 73.8% by day 5. Mean Hb and hematocrit levels were significantly lower in patients with severe leptospirosis compared to mild leptospirosis from days 3 to 10 (10-12 vs. 12-14g/dL; p<0.0001) |
| [[4](#_ENREF_4)] | 68 male dengue cases; 73 leptospirosis cases | | Prospective cohort study with 2-year follow-up: compared blood parameters of patients with leptospirosis and dengue fever | Inclusion criteria: positive dengue or *leptospira* IgM, immunoglobulin-M. Confirmed serologically: positive leptospira IgM | Thrombocytopenia <100,000/mm^3^ | | Thrombocytopenia in 47% of leptospirosis patients |
| [[44](#_ENREF_44)] | 207 male leptospiral positive; Brisbane | | Case-control study; compared Hb of patients infected with leptospiral serotypes from patient database | Isolated leptospires by PCR or serology with microscopic agglutination test (MAT) with >4-fold riser in titer | Hemoglobin | | Significant differences in Hb across the serotypes (F=2.67, p=0.004). *Canicola* and *Hardjo* (131.29 vs. 146.29 g/L, p=0.03); *Canicola* and *Robinsoni* (131.29 vs. 145.56 g/L, p=0.02); *Canicola* and *Tarassovi* (131.29 vs. 145.30 g/L, p=0.04); and *Canicola* and *Zanoni* (131.29 vs. 147.34 g/L, p=0.02) |
| [[45](#_ENREF_45)] | 239 leptospirosis cases: 12 severe, 227 uncomplicated | | Case-control study in Australia; blood samples were collected from patients with severe and uncomplicated leptospirosis to identify differentiating laboratory markers | Leptospirosis: confirmed by real-time PCR, or with >4-fold in MAT titer between presentation and convalescent follow-up, or MAT titer ≥400. Severe cases defined: respiratory distress, dyspnea, hemoptysis, diffuse alveolar hemorrhage, and/or acute liver or renal failure | Hb (135-185 g/L), hematocrit (0.39-0.52) | | Patients with severe leptospirosis had significantly lower Hb concentrations (mean ± SD; severe: 122.3 ± 0.66 vs. uncomplicated: 145.3 ± 0.90 g/dL; p=0.005), hematocrit (mean ± SD; severe: 0.36 ± 0.09 vs. uncomplicated: 0.43 ± 0.003 g/dL; p=0.003), platelet counts (mean ± SD; severe: 109.8 ± 20.2 vs. uncomplicated: 162.4 ± 3.8 x10^9^/L; p=0.03), and erythrocyte counts (mean ± SD; severe: 4.1 ± 0.02 vs. uncomplicated: 4.8 ± 0.03 x10^12^/L ; p=0.01), compared to uncomplicated leptospirosis cases. |
| [[47](#_ENREF_47)] | 42 patients with leptospirosis, 84 patients with dengue; Puerto Rico | | Case-control study; compared hematological status and symptoms in patients with leptospirosis and dengue fever | Leptospirosis: negative MAC-ELISA for dengue fever, positive for leptospirosis by IgM ELISA or MAT (titer >1:400) | Anemia: male: Hb<13.8, female: Hb<12.1 g/dL  Hyperbilirubinemia: bilirubin>1.3  Leukocytosis: WBC>10,0000  Thrombocytopenia: platelets<100,000 | | Jaundice, leukocytosis, hyperbilirubinemia, anemia, elevated creatinine, proteinuria, and hematuria were significantly associated with leptospirosis compared to dengue fever (p<0.01). 23 (85%) patients with leptospirosis had thrombocytopenia, 15 (71%) had hematuria, 19 (62%) had anemia, and 13 (48%) had leukocytosis |
| [[43](#_ENREF_43)] | 74 patients with leptospirosis; Iran | | Cross-sectional study; assessed hematological status in leptospirosis patients | Immunofluorescence antibody method (IFA) test for positive serology of leptospirosis. Anti-leptospira antibody titer >1/100 | Hematuria (> 3 RBC/HPF); anemia (male Hb <13, female Hb <12 g/dl); thrombocytopenia (platelets <150,000 cells/mm^3^) | | 87.3% had thrombocytopenia, 70.96% had hematuria, 15.5% had platelets less than 20,000/mm^3^, and 78.4% had anemia |
| [[46](#_ENREF_46)] | 34 patients with leptospirosis; France | | Retrospective cohort study; recorded hematological parameters and risk factors for confirmed leptospirosis patients | Leptospirosis: clinical symptoms, presence of antigen or class IgM antibody (ELISA) titer >1:400, and MAT titer >1:100 | Anemia: men: Hb<12 g/dL, women: Hb<11 g/dL | | Anemia (n=4), hematuria (n=6), platelet count <150,000/mm^3^ (n=6) |
| [[48](#_ENREF_48)] | 58 patients with leptospirosis; Moldova | | Retrospective cohort study; 5-year follow-up;  assessed frequency of clinical and laboratory symptoms | Leptospirosis: 4-fold increase in the initial ELISA titer or ≥-fold increase and acute renal failure (ARF, serum creatinine >150 mmol/L) | Hemolytic anemia: polychromatophilia, high unconjugated bilirubin, raised lactate dehydrogenase, reticulocytosis | | 72.4% hemolytic anemia  Mean Hb: 8.6 ± 2.7 g/dl |
| [[49](#_ENREF_49)] | 93 patients with leptospirosis; Korea | | Prospective cohort study; assessed hematological parameters of patients with acute febrile illnesses tested for leptospirosis | Leptospirosis: MAT titer >50% in serum dilution of 1:80 or 4-fold increase in titer in paired sera confirmed leptospirosis | n/a | | 37% had moderate to severe anemia, 22% had leukocytosis, 29% had leukopenia, 18% had thrombocytosis |

N/A, not applicable; micronutrient cutoffs not provided.
