# Supplementary material for: Micronutrients and Leptospirosis: A Review of the Current Evidence
Source: PLoS Negl Trop Dis. 2016 Jul 7;10(7):e0004652. doi: 10.1371/journal.pntd.0004652 (PMC4936698; doi:10.1371/journal.pntd.0004652)
Supplement: S2 Table — Calcium: Evidence from in vitro laboratory studies of the association between calcium and Leptospira infection. (DOCX) [file pntd.0004652.s002.docx]

**S2 Table. Calcium**

| **Authors** | **Sample (N)** | **Methods** | **Definition of Leptospirosis** | **Definition of Micronutrient** | **Main Findings** |
| --- | --- | --- | --- | --- | --- |
| ***Laboratory*** | | | | | |
| [[56](#_ENREF_56)] | Mouse macrophage line (J774A.1) and human macrophages (THP-1) | Ca^2+^ concentrations in infected macrophages were compared to uninfected macrophages. Cells were treated with Ca^2+^ chelators to determine source of Ca^2+^ | J774A.1 or THP-1 cells were infected with  *L. interrogans icterohaemorrhagiae* serotype *Lai* strain Lai at a density of 100 leptospires per cell | Changes in Ca^2^ calculated by: (fluorescence intensity in 500 infected cells) / (fluorescence intensity in the same number of cells before infection) x100% | *L. interrogans* infection significantly elevated Ca^2+^ levels (p<0.05) due to extracellular Ca^2+^ influx and intracellular Ca^2+^ release. High Ca^2+^ induced macrophage apoptosis and necrosis (p<0.05). Calcium chelators reduced the Ca^2+^ elevation during infection |
| [[57](#_ENREF_57)] | Human kidney cells *in vitro* | Ca^2+^-binding mutants of LipL32 (lipoprotein 32) were created to analyze the role of the Ca^2+^-binding cluster in LipL32 | *L. shermani* Lipl32 gene transformed into *E. coli* | Ca^2+^ binding integrity to LipL32 assessed by CD (circular dichroism) spectrometry using a probe for detecting Ca^2+^ -binding proteins | Ca^2+^ binding of LipL32 essential for regulating interaction with TLR2 (toll-like receptor 2) for inflammatory response induction |
| [[58](#_ENREF_58)] | *E. coli* strain B834 | CD spectra assessed binding affinity of LipL32 to Ca^2+^ and fibronectin F30 (human fibronectin fragment) in *E. coli* | *L. shermani* LipL32 gene transformed into *E. coli* | Ca^2+^ and fibronectin F30 binding affinity assessed by CD spectrometry | Ca^2+^ promotes LipL32 binding to fibronectin. Binding affinity for F30 was stronger for Ca^2+^ bound LipL32 than for Ca^2+^-free LipL32 (K_d_ values = 0.29 ± 0.01 μM and 1.15 ± 0.06 μM, respectively; p<0.0001, R^2^=0.99) |
| [[59](#_ENREF_59)] | *E. coli* BL21 | LipL32 mutants (D163-168A, Q67A, and S247A) were created to assess affinity for Ca^2+^ and human plasminogen and fibronectin | LipL32 gene isolated and transformed into *E. coli* cells | Binding affinity to Ca^2+^ and to human plasminogen/fibronectin assessed | Wild type and mutant LipL32 bound to plasminogen and fibronectin with similar affinities both with and without Ca^2+^; Ca^2+^ not required for interaction between LipL32 and host extracellular matrix proteins |
| [[89](#_ENREF_89)] | Isolated Lig proteins | Lig proteins isolated to assess binding affinity to Ca^2+^ and to fibronectin | Lig proteins isolated | Effect of Ca^2+^ on binding affinity of Lig proteins to fibronectin | Ca^2+^ binding increases conformational stability of LigBCen2 (binds to host extracellular matrix proteins) Midpoint of LigBCen2 unfolding increased from 50.7 ± 0.9 to 54.8 ± 0.5 ºC when Ca^2+^ was added.  Ca^2+^ increases binding affinity (K_d_ = 63 nM compared to apoprotein K_d_ = 272 nM) |

N/A, not applicable; micronutrient cutoffs not provided.
