# Supplementary material for: Micronutrients and Leptospirosis: A Review of the Current Evidence
Source: PLoS Negl Trop Dis. 2016 Jul 7;10(7):e0004652. doi: 10.1371/journal.pntd.0004652 (PMC4936698; doi:10.1371/journal.pntd.0004652)
Supplement: S3 Table — Trace minerals: Evidence from in vitro, animal, and human studies of the association between trace minerals and Leptospira infection. (DOCX) [file pntd.0004652.s003.docx]

**S3 Table. Trace Minerals**

| **Authors** | **Sample (N)** | **Methods** | **Definition of Leptospirosis** | **Definition of Micronutrient** | **Main Findings** |
| --- | --- | --- | --- | --- | --- |
| ***Laboratory*** | | | | | |
| [[62](#_ENREF_62)] | 2x10^9^ leptospires per mL | Gelatinase dependence on metal ions was evaluated by extracting gelatinase via centrifugation and incubating with each cation | *L. interrogans* serotype *Icterohaemorrhagiae* strain RGA | CaCl_2_, ZnCl_2_, CuSO_4_, MgSO_4_, and FeCl_2_ each at a concentration of 0-100 μM | 100 mM of metal chelators EDTA and EGTA caused 60-70% inhibition of gelatinase. Cu^2+^, Mg^2+^, Mn^2+^, and Zn^2+^ increased activity of gelatinase (p<0.05). Fe^2+^ increased inhibition by 40% (p<0.05) |
| [[63](#_ENREF_63)] | 2x10^6^ leptospires per mL | Compared function of leptospiral ABC (ATP-binding cassette transporter) ATPase and Mn^2+^ utilization in EMJH and iron-deficient EMJH with 100 μM MnCl_2_ to evaluate leptospira ability to grow with a cation other than Fe^2+^ | *L. biflexa* serotype *Patoc* strain Patoc 1, L. *interrogans* serotype *Manilae* strain L495, and *L. interrogans* serotype *Copenhageni* strain Fiocruz | EMJH medium with 180 μM FeSO_4_ and 5 μM ZnCl_2_. Treatment: FeSO_4_ replaced with 100 μM MnCl_2_. % survival: ratio of number of CFU (colony-forming unit) for cells incubated with MnCl_2_ to that for cells without MnCl_2_ | Inactivation of ATPase-coding gene prevented leptospire growth in Mn^2+^ instead of Fe^2+^. Wild type growth unaffected (100%) but a 50% reduction in LEPBla2866 mutant growth (p=0.05) in medium with Mn^2+^ instead of Fe^2+^ |
| [[65](#_ENREF_65)] | 2x10^6^ leptospires per mL | Cultures grown with varying levels of trace minerals to determine concentrations for leptospiral growth | 10 serotypes of *L. interrogans.* 4 serotypes of *L. biflexa* | 0.2 x10^-6^g/mL Zn(NO_3_)_2_  1x10^-9^ g/mL CoSO_4_  1x10^-6^ g/mL CuSO_4_ | Low Zn^2+^ and Co^2+^ concentrations stimulated leptospiral growth; high concentrations inhibited growth. Low Cu^2+^ stimulated growth of saprophytic but not pathogenic. |
| [[66](#_ENREF_66)] | 0.5–4.5x10^6^ leptospires per mL | Effect of Zn on growth was evaluated by adding straisn to 5 mL Korthof medium either without zinc nitrate (control) or with zinc nitrate (treatment).  Growth determined by measuring OD | *L. icterohaemorrhagiae*  *L. naam Naam*  *L. schuffneri Vleermuis*  *L. broomi Patane*  *L. pyrogenes Salinem*  *L. cynopteri Vleermuis*  *L. mini Szwajizak*  *L. medanensis Hond*  *L. bataviae Swart* | Korthof medium supplemented with 2x10^-7^ g Zn(NO_3_)_2_•6H_2_O/mL | Zinc nitrate significantly increased growth for each strain. OD of *L. naam Naam* increased from 0.38 to 0.49, t=15.07, p=0.001 |
| [[68](#_ENREF_68)] | 10^7^ -4.0 x10^7^ leptospires per mL | Mineral growth requirements were determined by testing different media, each prepared without the cation being tested | *L. canicola* strain Dog-L-Reinhard | n/a | Ca^2+^ required for growth unless replaced by Ba^2+^ or Sr^2+^. Thiamine and iron were required for growth.  Low magnesium (20-80 mg/100 mL) inhibited growth; lack of magnesium prevented growth.  Addition of vitamin B_12_ (0.0002 ug/mL) or biotin (0.02 ug/mL) increased growth to 3x10^7^ cells/mL |
| [[67](#_ENREF_67)] | 4x10^7^ leptospires per mL | Ca^2+^, Mg^2+^, and K^+^ were studied as growth factors for leptospira using phonephelometer to measure growth | *L. Pomona* | 3x10^-5^ M Ca^2+^  5x10^-6^ M Mg^2+^  5x10^-5^ M K^+^  EDTA | Ca^2+^ and magnesium combined stimulated growth. Mg^2+^ deficiency (<5x10^-7^ M) inhibited growth. Potassium stimulated growth. 10^-4^ M concentration of divalent metal cation required for growth |
| ***Animal*** | | | | | |
| [[70](#_ENREF_70)] | 225 free-ranging sea lions | Prevalence of *L. interrogans* and sensitivity of diagnostic test evaluated. Serum samples measured for Ca^2+^, phosphorus, K^+^ to assess association with infection | *L. interrogans* serotype *Pomona* diagnosed by MAT | Serum calcium, phosphorus, potassium, and sodium measured | Sea lions with phosphorus >7mg/dL were 6.8 times more likely of being infected with serotype *Pomona* compared to those with phosphorus ≤7mg/dL (OR: 6.8, 95% CI: 2.4-20.3, p<0.05). Sea lions with calcium >8.9 mg/dL were 16.8 times more likely of being infected with serotype *Pomona* compared to those with lower calcium levels (OR: 16.8, 95% CI: 4.3-70.0, p<0.05). No significant associations between *Leptospira* infection and potassium (OR: 2.3, 95% CI: 0.8-6.1) or sodium (OR: 1.7, 95% CI: 0.7-4.3) levels |
| [[71](#_ENREF_71)] | 13 Grivet monkeys, 6 infected with balcanica and 6 with tarassovi, 5.1x10^8^ leptospires/mL | Monkeys were injected with either leptospirosis strain. Serum Zn and Fe concentrations were measured from blood samples | Exposure: *L. interrogans* serotypes *balcanica* and *tarassovi* | Serum zinc and iron concentrations | Zn^2+^ concentrations did not change. Serum Fe^2+^ levels did not change in most animals but decreased in 2 monkeys 4-7 days after infection. Trace metals were not significantly different in either strain |
| [[72](#_ENREF_72)] | Gold Syrian hamsters (40-80g) | Hamsters inoculated with *L. canicola Moulton* cloned three times to develop strains of high and low virulence from the same serotype. | *L. Pomona* Pomona and *L. canicola* Moulton | Medium contained 8.5 mM NaCl, 5.4 mM KCl, 0.4 mM MgSO_4_, 0.34 mM CaCl_2_, 1.0 mM NH_4_CL, 0.1 mM Na_2_HPO_4_, 1.0 mg/L thiamine, 2 μg/liter Vitamin B12 | Virulent strain required at least 0.001 ug/mL biotin for growth. Mg^2+^ stimulated virulent strain growth, but not avirulent strain growth |
| [[73](#_ENREF_73)] | Mature golden hamsters, 100 g each, infected with 3 x10^6^ leptospires | Hamsters inoculated with leptospiral strains and treated with selenium compounds 18 to 24 hours after infection, once daily for 5 days. After 30 days, hamsters were euthanized and kidneys were removed for analysis | *L. australis, L. autumnalis, L. bataviae, L. canicola, L. grippotyphosa, L. icterohaemorrhagiae*, and *L. Pomona* | Selenium compounds: 2-Acetamido-4-methoxy-phenyl diselenide, selenourea, selenium disulfide, selenium diethyl dithiocarbamate, p-(phenylselenyl) aniline, Phenyl diselenide, Phenyl selenide | Synthetic organic compound of selenium inhibited leptospiral growth. 5 mg/kg dose of Selenourea inhibited growth against all strains except *L. canicola* and *L. icterohaemorrhagiae* |
| ***Human*** | | | | | |
| **Authors** | **Sample (N)** | **Methods** | **Definition of Leptospirosis** | **Definition of Micronutrient** | **Main Findings** |
| [[74](#_ENREF_74)] | 15 patients with severe leptospirosis; Australia | Retrospective cohort study; measured serum magnesium concentrations over 10 days in patients with severe leptospirosis | Severe leptospirosis: respiratory hemorrhaging and/or acute liver or renal failure | Hypomagnesemia: <0.70 mmol Mg^2+^/L serum | 14 of the 15 patients had hypomagnesemia during the 10 days . Serum magnesium concentrations ranged from 0.48-0.69 mmol Mg^2+^/L |
| [[61](#_ENREF_61)] | 20 patients with leptospirosis; Thailand | Prospective cohort study; evaluated renal function and Mg^2+^ levels in patients. Urine and blood Cr, Ca^2+^, Mg^2+^, and phosphorus were measured; fractional excretion of Mg^2+^and tubular reabsorption of P were calculated. | Patients were admitted to hospital for leptospirosis | Hypokalemia (<3mmol/L); hypocalcemia (<2 mmol/L); hypophosphatemia (<0.9 mmol/L); hypomagnesemia (0.7 mmol/L). Normal fractional excretion of magnesium <2.2% | 15 patients (75%) had renal magnesium wasting; 10 patients (50%) had hypomagnesaemia; 4 patients (20%) had hypophosphatemia and hypokalemia; 5 patients (25%) had hypocalcemia. Median fractional excretion of Mg was significantly greater for patients with ARF (10.1%) than for those without ARF (3.1%) (p<0.01) |

N/A, not applicable; micronutrient cutoffs not provided.
