# Supplementary material for: Micronutrients and Leptospirosis: A Review of the Current Evidence
Source: PLoS Negl Trop Dis. 2016 Jul 7;10(7):e0004652. doi: 10.1371/journal.pntd.0004652 (PMC4936698; doi:10.1371/journal.pntd.0004652)
Supplement: S4 Table — B-vitamins: Evidence from in vitro and animal laboratory studies of the association between B-vitamins and Leptospira infection. (DOCX) [file pntd.0004652.s004.docx]

**S4 Table. B-Vitamins**

| **Authors** | **Sample (N)** | **Methods** | **Definition of Leptospirosis** | **Definition of Micronutrient** | **Main Findings** |
| --- | --- | --- | --- | --- | --- |
| ***Laboratory*** | | | | | |
| [[79](#_ENREF_79)] | 3x10^8^-4x10^8^ leptospires/mL | Vitamin requirements were determined by omitting each nutrient from the medium. Culture growth was measured as change in % light transmittance relative to control tube. | *L. Pomona* strain T-262 | Vitamin B_12_: 0.000123 mg per 100 mL. Thiamine: 0.0558 mg per 100 mL | Thiamine and vitamin B12 deletions reduced growth.  Vitamin B_12_ and thiamine only two nutrients required for adequate growth |
| [[80](#_ENREF_80)] | 5x10^3^-2x10^4^ leptospires/mL | Dark-field microscope measured growth of leptospires in optical fields | *L. canicola, L. Pomona, L. grippotyphosa* | 1 mL of trace elements were added: 37.5 mg/L Zn^2+^, 3.75 mg/L Mn^2+^, and 2.5 mg/L Cu^2+^ to 100 mL of standard medium with vitamin B_12_ and thiamine | Growth was stimulated by thiamine, vitamin B_12_, potassium, and calcium ions  Vitamin B_12_ was essential for growth |
| [[81](#_ENREF_81)] | N/A | Cultures were grown in vitro and tested with medium to determine nutrient requirements | *L. Pomona* and 13 other serotypes | 160 μg vitamin B_12_  160 μg thiamine | Vitamin B_12_ was required for growth |
| [[82](#_ENREF_82)] | 2x10^6^-3x10^6^ leptospires/mL | Different media were tested to determine nutritional requirements for leptospires with vitamins added | *L. Pomona* strain Wickard | 5 μg/mL concentration of vitamins were added to basal medium | Thiamine was the only vitamin required for growth and produced 103 nephelometer reading; vitamin B_12_ only produced 16 nephelometer reading |
| [[83](#_ENREF_83)] | n/a | Leptospire growth tested with vitamin B_12_ as a growth factor | n/a | Korthof medium with varying concentrations of vitamin B_12_ and 1 ϒ/mL nicotinic acid | Vitamin B_12_ concentrations were associated with increased growth. Opacimetric values: 3.2 for no Vitamin B_12_, 3.8 at 0.01 ϒ/mL Vitamin B_12_, and 4.3 for 0.1 ϒ /mL vitamin B_12_ |
| [[84](#_ENREF_84)] | n/a | Nutritional requirements were evaluated by testing the medium supplemented with vitamins | *L. canicola, L. Pomona, L. ballum, L. scjroe, L. icterohaemorrhagiae* | n/a | Biotin, riboflavin, pyridoxine, thiamine, nicotinic acid, and folic acid increased optical density (growth). Thiamine was the only essential vitamin |
| ***Animal*** | | | | | |
| [[85](#_ENREF_85)] | 10 hamsters;  2.7x10^6^ cells/hamster | *L. hardjo* cultured in media with range of vitamin B_12_ to determine the effects on growth. Hamsters were inoculated and euthanized, and kidney, brain, liver, and blood isolates were tested through 5 subcultures in vitamin B_12_ and thiamine depleted medium | *L. hardjo* | 0.16 - 0.16x10^-9^ μg vitamin B_12_ /mL | Vitamin B_12_ was associated with increased growth. Minimal vitamin B_12_ requirement for growth was established (0.16x10^-4^ – 0.16x10^-9^ ug/mL). Hamster isolates experienced reduced growth in media lacking vitamin B_12_ and thiamine. |
| [[86](#_ENREF_86)] | Hamsters injected with 2.6x10^3^ leptospires | Cultures were grown in medium with varying component deletions. Virulence tests were conducted in hamsters. Hamsters were euthanized and kidneys obtained for analysis. | *L. interrogans* serotype *canicola* | P-80 medium with thiamine and vitamin B_12_ deletions | Vitamin B_12_ deletion inhibited growth to a nephelometer reading of zero. Thiamine deletion reduced growth, but not to zero. Nephelometer reading reduced from 49 at 26x10^5^ canicola cells/mL to zero at 26x10^3^ cells/mL |

N/A, not applicable; micronutrient cutoffs not provided.
